# Tissue Regeneration and Tumour Development

**DOI:** 10.1038/bjc.1959.74

**Published:** 1959-12

**Authors:** J. O. Laws

## Abstract

**Images:**


### TISSUE REGENERATION AND TUMOUR DEVELOPMENT

J. O. LAWS

*From the Department of Experimental Pathology and Cancer Research, School of Medicine, Leeds, 2*

Received for publication October 2, 1959

THE early changes, including hyperplasia, induced in the liver of the rat by the oral administration of 2-acetylaminofluorene were described by the author and his French colleagues (Laws, Mabille, Royer and Rudali, 1952) and in less detail by Skoryna and Webster (1951). The studies have been continued for some years in this department. Recently Laird and Barton (1959) have investigated the quantitative aspect of this effect and have pointed out that the period up to the onset of hyperplastic change in the liver of animals treated with this carcinogen appears to coincide with the minimum period of administration of the chemical which is necessary to provoke the ultimate appearance of tumours in this organ. The work described here has made use of the possibility of inducing regenerative hyperplasia in the liver by means of partial hepatectomy to study in more detail the influence of such a reaction on the effect of 2-acetylaminofluorene. Histological examination of the liver during the process has helped to make clear some of the mechanisms involved.

### MATERIALS AND METHODS

*Animals.*—The rats used in most of this work were bred in this laboratory and were of Wistar stock. In the experiment contrasting the behaviour of young and old rats, the animals were of Birmingham stock, obtained from the Department of Anatomy, University of Birmingham. No difference has been noticed in the response of these two stocks of rats to treatment with 2-acetylaminofluorene. Only male rats were used throughout, since liver changes are rarely found in females. Unless noted all rats were adult (weight at least 200 g.) when the experiments began.

*Carcinogen treatment.*—The carcinogen, 2-acetylaminofluorene was obtained in pure form from L. Light and Sons. It was used, at a strength of 0·1 per cent incorporated in a meal diet supplied by the North Eastern Agricultural Cooperative Society, Aberdeen. The carcinogen was dissolved in 500 ml. of acetone and mixed with 10 kg. of the meal which became lightly dampened throughout. Water was added to form a paste which was formed into cakes and dried. These were fed *ad libitum* to the rats together with water. The meal used was of the same composition as the rat cakes from the same manufacturers used to feed the general stock of rats and mice, and provided a full balanced diet.

*Partial hepatectomy.*—The operation, by the method of Higgins and Anderson (1931), consisted of the removal of the anterior and left anterior lobes of the liver after ligature. Approximately two-thirds of the liver substance was effectively removed, on the average.

*General.*—All animals included in the experimental figures were subjected to a full post-mortem examination and histological sections of all relevant lesions were examined after staining with haematoxylin and eosin.

### RESULTS

The earliest changes provoked by 2-acetylaminofluorene are of a toxic nature, reducing the efficiency of the liver cells and leading to the death of many cells. In earlier experiments of the author (Laws *et. al.*, 1952) and in those of Laird and Barton (1959) this was in some cases sufficient to lead to the death of the animals. Even in those rats less severely affected, histological examination of the liver showed a loss of parenchymatous cells around the portal tracts, with hyperplasia of small non-parenchymatous cells of uncertain origin. This stage is followed by one in which cell division among the parenchyma cells rapidly produces a replacement of the lost tissue, and goes on steadily to genuine hyperplasia. In the animals used in the present experiments (albino rats of so-called Wistar strain bred in the laboratory, but of uncertain ancestry), the changes are less dramatic, death seldom occurring during normal 2-acetylaminofluorene feeding. Histologically also the changes are more gradual but the outcome is the same, a nodular, hyperplastic liver. In spite of the little apparent change in the livers of these animals, partial hepatectomy performed after three weeks feeding unmasks a profound diminution in liver efficiency. If such animals are treated with the carcinogen after the operation also, most of them are dead within two weeks (26 out of 40 animals up to date). Such animals which die or are killed at this time are found to have liver weights only about one-third of the normal, i.e. only about the amount of liver left by the hepatectomy, in contrast with the rapid replacement of liver tissue in normal animals after such an operation. Ascites is invariably present and usually other effusions and a general waterlogging of the tissues. This probably results from hypoalbuminaemia due to poor liver function, although this has not so far been proved by direct estimation.

Histologically such livers present the series of changes noted above in a very severe form. Within a few days of the operation much of the parenchyma of the remaining lobes has disappeared, principally around the portal tracts, and there is marked proliferation of small cells in this region, stretching out into the parenchyma which is still present (Fig. 3). If the animal survives as long as a week, regeneration begins and can be seen by the eighth day by the naked eye in the form of small translucent nodules on the surface of the organ, and on the cut surfaces. Such nodules can be shown by a series of preparations examined histologically to originate as small groups of cells (presumably formed in the first place by division of one cell) staining basophilically and containing many mitotic figures (Fig. 1 and 2). They are always situated in the region of the radicles of the hepatic vein, the part least damaged, although they enlarge rapidly and by the fourteenth day may largely have replaced the remaining old parenchymatous cells, and the small cells which had infiltrated (Fig. 4). The animal may die even at this stage but if it survives, the enlargement continues to reproduce the original bulk of the liver and beyond this to genuine hyperplasia.

Hepatectomy performed five weeks after the commencement of carcinogen feeding leads to similar results if the administration is continued after operation. After seven weeks of feeding however the mortality is much less, and the animals remain healthier. Liver regeneration is still slow in such animals, but the organ weight reaches the lower limits of normal in some by the fourteenth day. Microscopically such animals show less destruction after operation and a more rapid onset of the nodular regeneration, otherwise similar in type. At the other extreme, if hepatectomy is performed on the day on which feeding with 2-acetylaminofluorene is started there is little delay in the onset of a normal general regenerative reaction, and the liver weight returns to near the normal limits in seven days. There is however the tendency for the weight to remain a little below normal, and nodules start to form as in the other cases. By the fourteenth day such nodules containing many mitotic figures are prominent. Regeneration in animals given three weeks treatment with carcinogen and then two weeks on a normal diet before hepatectomy behave much like those given no treatment before operation. This suggests that an extreme degree of liver damage, likely to cause death after hepatectomy, is due to cumulative toxic action on the liver cells by the 2-acetylaminofluorene. Such an extreme degree of damage is not a prerequisite for the onset of nodular proliferation, although this process is undoubtedly more vigorous in animals threatened with imminent decease from liver failure. It does appear that some degree of " strain " precipitated by functional incapacity, is needed to start this process going. How such a " strain " can be translated into a stimulus which results in the production of a new race of liver cells capable of resisting the toxic effects of 2-acetylaminofluorene, and of still responding to the physiological stimulus for regeneration, remains to be investigated.

So many of the changes which accompany carcinogenesis in any organ must be incidental that to establish a connection between such events and the process of carcinogenesis itself it is essential to show that they do affect the actual production of tumours. In the case under consideration the performance of a hepatectomy at the appropriate period might be expected to alter the latent period or the incidence of liver tumours. It might also shorten the period of administration of carcinogen needed to produce tumours, if the precocious appearance of the changes described, means that the " initial, essential step " (Laird and Barton, 1959), has already been accomplished at the end of three or four weeks. Experiments already carried out by the author (Laws, 1956) have established an effect of hepatectomy, carried out at the beginning of the carcinogen feeding period, on the latent period of liver tumour production, although the incidence of tumours was unaltered. In the first experiment (Table I), animals treated by this pro-

TABLE I

| Group | Hepatectomy performed | Hepatoma incidence in periods (after start of AAF feeding): (a) 12–14 months | (b) 15–17 months | (c) 18–24 months | Total |
|---|---|---|---|---|---|
| A | None (normal controls) | 1 | 6 | 1 | 8/11 |
| B | 3 weeks before AAF feeding started (Hepatect. controls) | 0 | 5 | 3 | 8/10 |
| C | At start of AAF feeding | 5 | 4 | 1 | 10/10 |
| D | After 3 months of AAF feeding | 0 | 1 | 8 | 9/11 |
| E | At end of AAF feeding (4 months) | 1 | 3 | 4 | 8/10 |

(Total out of number alive at twelve months.)

cedure showed five deaths with gross liver tumours out of ten animals at a time when only two out of forty-two other rats not hepatectomised early in the feeding period had developed tumours. These other animals included, as well as normal controls, animals hepatectomised after three and four months of carcinogen feeding, that is to say after the onset of hyperplasia. In these latter animals the onset of tumours was, if anything, delayed beyond the period seen in the normals. Skoryna and Webster (1951) also concluded that partial hepatectomies started in the third month of 2-acetylaminofluorene feeding, even if repeated, did not accelerate the appearance of tumours. A further experiment on these lines is still in progress ; the first results are similar to those of the earlier one.

The period by which the appearance of tumours was accelerated was about three months, which coincides roughly with the period taken in our animals for the appearance of hyperplasia on plain 2-acetylaminofluorene feeding. This would fit in with the suggestion of Laird and Barton (1959) that it is from the onset of hyperplasia that the irreversible process of tumour formation should be dated. It would seem that the latent period before the onset of rapid tumour growth is unaffected by hepatectomy outside the " critical " period, which lasts up to the time of onset of hyperplasia during 2-acetylaminofluorene feeding. The finding of Glinos, Bucher and Aub (1951) that in rats treated with dimethylaminobenzene, hepatectomy at the time of withdrawal of the carcinogen appeared slightly to hasten the appearance of tumours, suggests that the mechanism of action of the azo-dye carcinogens may differ from that of 2-acetylaminofluorene. This is made more probable by the difference in the time-course of liver hyperplasia noted by Laird and Barton when using a carcinogen of the azo-dye group (3′-methyl-dimethylaminoazobenzene).

These authors also note that the onset of hyperplasia occurred later in rats which were of greater weight, and incidentally older, at the time carcinogen feeding was started. In another experiment carried out by the author in this laboratory it has been shown that the latent period before the onset of active tumour growth is also affected by the age of the rats at the time at which feeding with carcinogen is started. Two groups of rats were given the same course of four months feeding with 2-acetylaminofluorene. The first group had an average body weight of 100 g. and the second an average of 380 g. at the start. The small rats gained weight during the four months in spite of treatment, the larger animals lost weight. The results, not previously reported, show a significantly earlier incidence of tumours in the young group (Table II). By fourteen months there had been six

---

EXPLANATION OF PLATE

FIG. 1.—Rat 1. Wt. 260 g. Wt. of liver at death 4·5 g. (normal 10 g.). Killed 8 days after partial hepatectomy. Infiltration of cells round portal tracts and basophilic nodules near central veins. × 110.

FIG. 2.—Rat 1. High power view of basophilic nodule with mitosis. × 550.

FIG. 3.—Rat 2. Wt. 260 g. Wt. of liver at death 2·75 g. (normal 10 g.). Found dying nine days after partial hepatectomy. Severe infiltration round portal areas with diminution of parenchyma. Basophilic nodules only beginning to form. × 65.

FIG. 4.—Rat 3. Wt. 360 g. Wt. of liver at death 3·5 g. (normal 13 g.). Found dying 14 days after partial hepatectomy. Liver contained many pale or translucent areas on naked-eye examination. Nodules have enlarged and are compressing the surrounding original tissue and the infiltrating cells. × 65.

(All animals received carcinogen treatment for three weeks before hepatectomy, and subsequently until death.)

1

2

3

4

Laws.

TABLE II

| Group | Hepatoma incidence in periods (after start of AAF feeding): (a) 12–14 months | (b) 15–16 months | (c) 17 months and over | Total |
|---|---|---|---|---|
| Young (100 g. body wt.) | 6/7 (i.e. 1 death without hepatoma) | 0/1 | 2/2 | 8/10 |
| Old (380 g. body wt.) | 1/4 (i.e. 3 deaths without hepatoma) | 0/3 | 3/4 | 4/11 |

(Hepatoma deaths given as fraction of total deaths.)

deaths with large liver tumours in this group, but only one in the older animals. Later tumours appeared more frequently in the older group. Comparison of the total tumour incidence in the two groups is unsatisfactory since many of the older animals died before reaching the period of highest tumour incidence in this group. There was a difference of about ten weeks in the median tumour death ages for the two groups, which is similar to the difference in the time of onset of hyperplasia noted by Laird and Barton in their experiments with groups of rats of different initial weights, using this carcinogen.

### DISCUSSION

Following partial hepatectomy in rats which have received 2-acetyl-aminofluorene, mitotic activity occurs only in the hyperplastic nodules which then appear. Even when the nodules are still small, mitoses have not been seen in the sickly parenchyma around them. Once a nodule has formed mitosis occurs rapidly, and the eventual hyperplastic liver has apparently been formed from the descendants of the comparatively small number of cells which have originated the nodules. This process contrasts with the diffuse mitotic activity seen in normal regeneration, and which is reported by Laird and Barton (1959) as occurring in rats treated with 3-methyl-dimethylaminoazobenzene. For this reason it would seem unwise to compare directly results obtained with the two carcinogens, or to assume that quantitative results obtained with one can be applied to a consideration of the mode of action of the other. Under the conditions of nodular replacement obtaining in the 2-acetylaminofluorene treated animals, regenerative activity is of course slower. The time taken in the experiments for the return of the liver weight to normal levels is sufficiently slow to allow for the extra generations of cells which must be produced. Observations on the similar, though more gradual formation which occurs in uncomplicated carcinogen feeding suggests that here too there is a gradual replacement of " normal " liver cells by the descendants of a small number of cells which have been changed in some way by exposure to the carcinogen. In particular, as suggested by Skoryna and Webster (1951), they appear to have become resistant to the toxic action. In spite of this suggestion that all the cells in a hyperplastic liver are " changed ", tumours arise from only a minority of the cells by a further, later burst of mitotic activity accompanied by other changes in behaviour. It seems that as Laird and Barton (1959) suggest, an essential step for carcinogenesis occurs when hyperplasia supervenes. With 2-acetylaminofluorene further alterations, perhaps not dependent on the direct action of the carcinogen, are necessary before true tumour formation can occur, as well as subsequently. Further the possibility must be considered that the change in certain cells which results in a resistance to the toxic action of the carcinogen and which enables hyperplasia to occur, may not necessarily be the essential carcinogenic change. It may accompany this, or set the stage for it. Experiments at present in progress on the behaviour of cells isolated from hyperplastic livers into tissue culture may help to throw some further light on the changes which occur at this stage, as may others in which a short period of carcinogen feeding, too short to give rise to tumours normally, is being combined with hepatectomy at the second or third week of feeding.

### SUMMARY

(1) Liver regeneration in rats following hepatectomy is severely delayed and disorganised when the carcinogen 2-acetylaminofluorene is administered for three weeks before the operation and subsequently. Regeneration under these circumstances occurs by nodular proliferation from a relatively small number of centres.

(2) When hepatectomy is performed at the beginning of the carcinogen feeding period, the initial phase of regeneration proceeds normally but is quickly superseded by nodular replacement. Such animals show a reduction in the latent period for liver carcinogenesis.

(3) Rats started on a course of 2-acetylaminofluorene treatment at the age of eight weeks also show a reduced latent period for liver carcinogenesis as compared with rats which are adult at the start of the treatment.

(4) It is suggested that during such treatment with 2-acetylaminofluorene an irreversible step in the process of carcinogenesis occurs at the onset of nodular hyperplasia. Hepatectomy early in the feeding period probably brings this step about precociously.

It is a pleasure to acknowledge the technical assistance of Miss Sallie Yates, and the help of Mr. C. Eastcott with the photomicrographs.

### REFERENCES

GLINOS, A. D., BUCHER, N. L. R. AND AUB, J. C.—(1951) *J. exp. Med.*, **93**, 313.
HIGGINS, G. M. AND ANDERSON, R. M.—(1931) *Arch. Path.*, **12**, 186.
LAIRD, ANNA K. AND BARTON, A. D.—(1959) *Nature, Lond.*, **183**, 1655.
LAWS, J. O.—(1956) Rep. Brit. Emp. Cancer Campgn., **34**, 301.
*Idem*, MABILLE, P., ROYER, R. AND RUDALI, G.—(1952) *Bull. Ass. franç. Cancer*, **39**, 450.
SKORYNA, S. C. AND WEBSTER, D. R.—(1951) *Proc. Soc. exp. Biol., N.Y.*, **78**, 62.


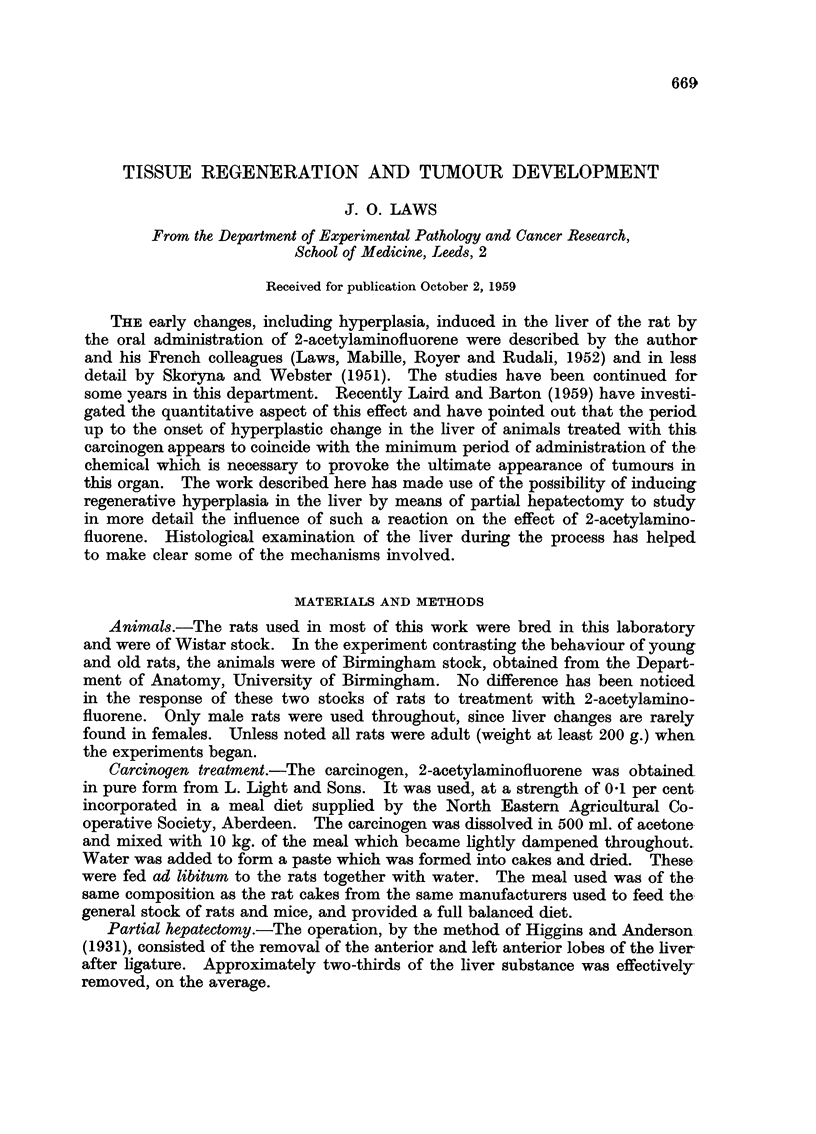

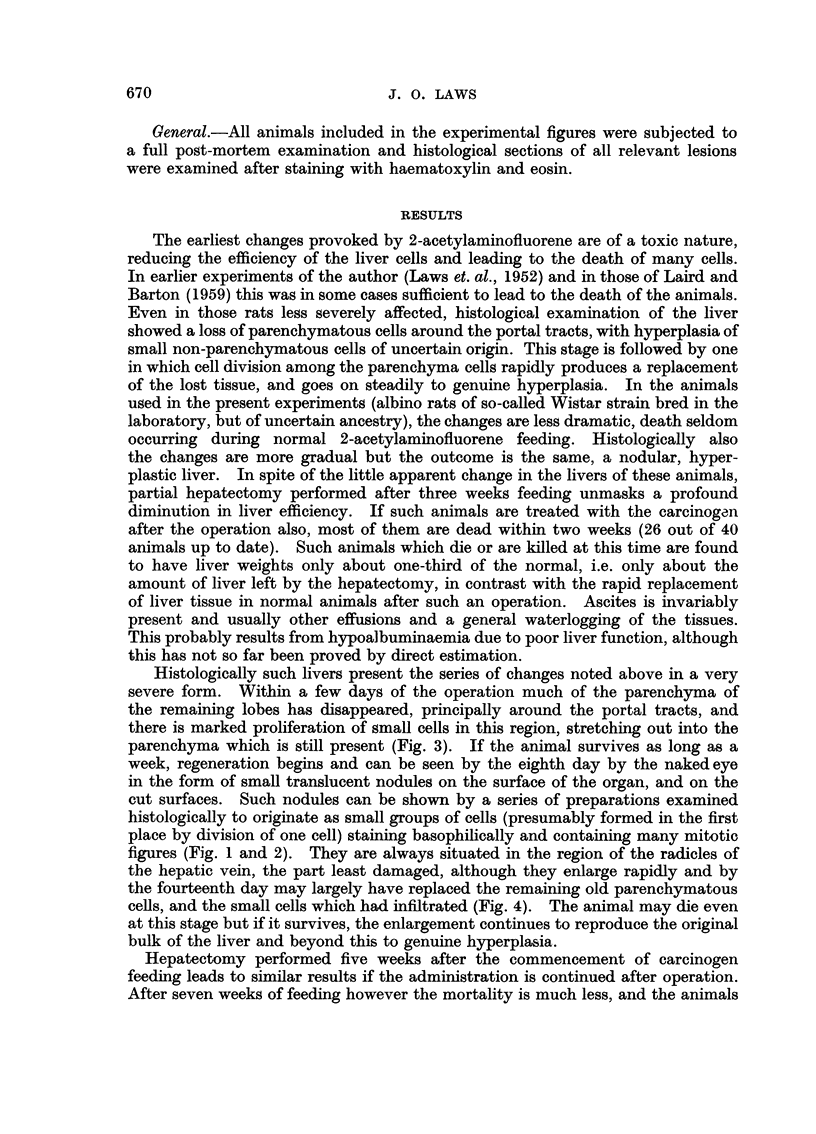

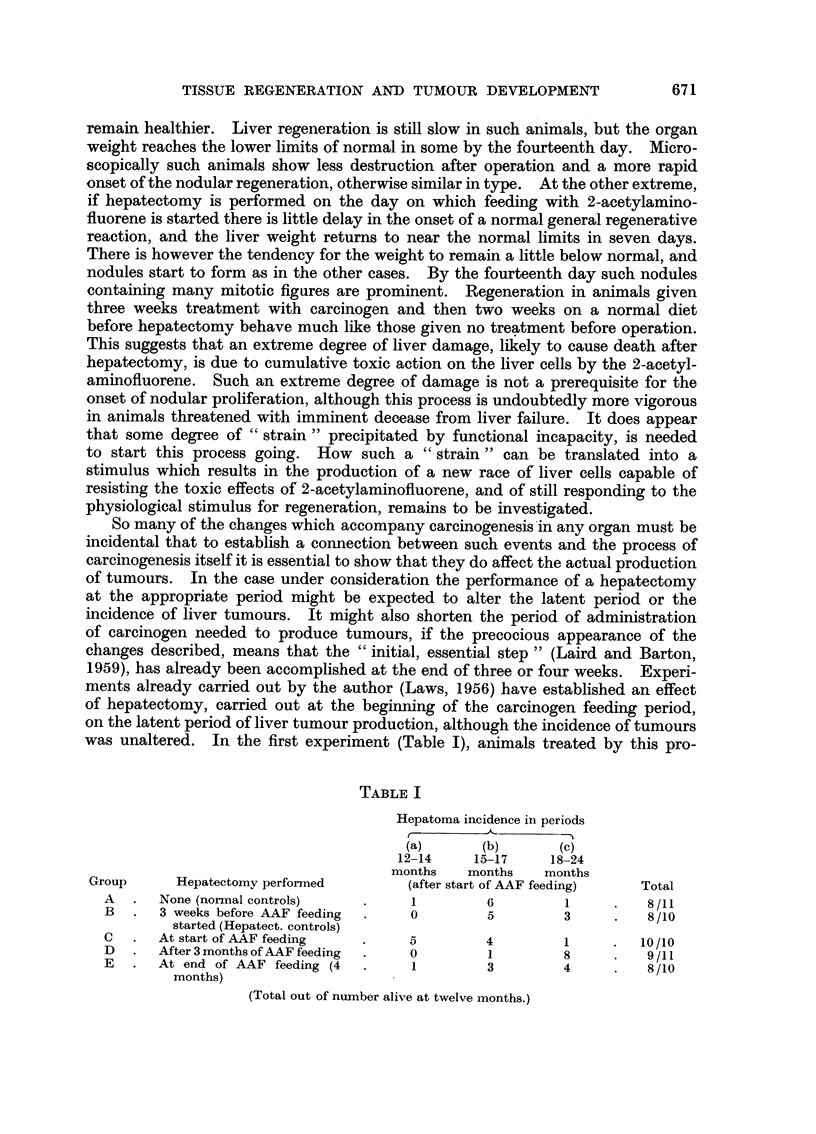

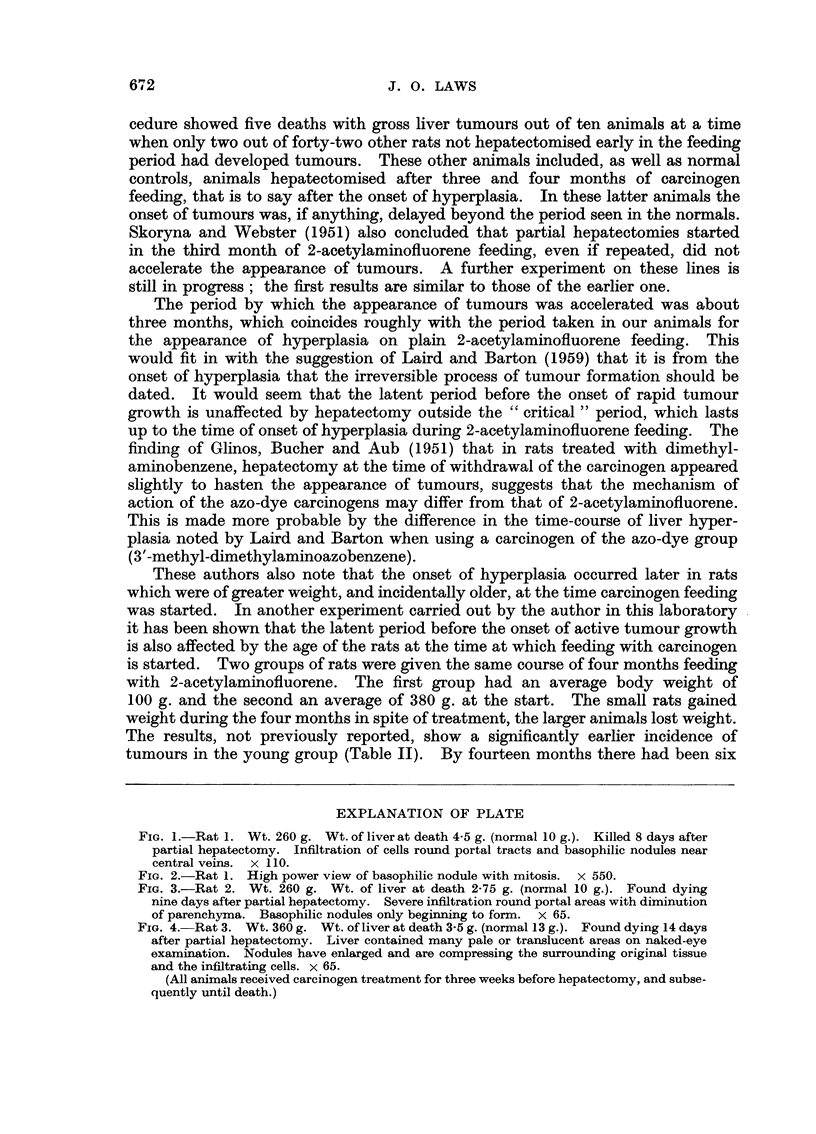

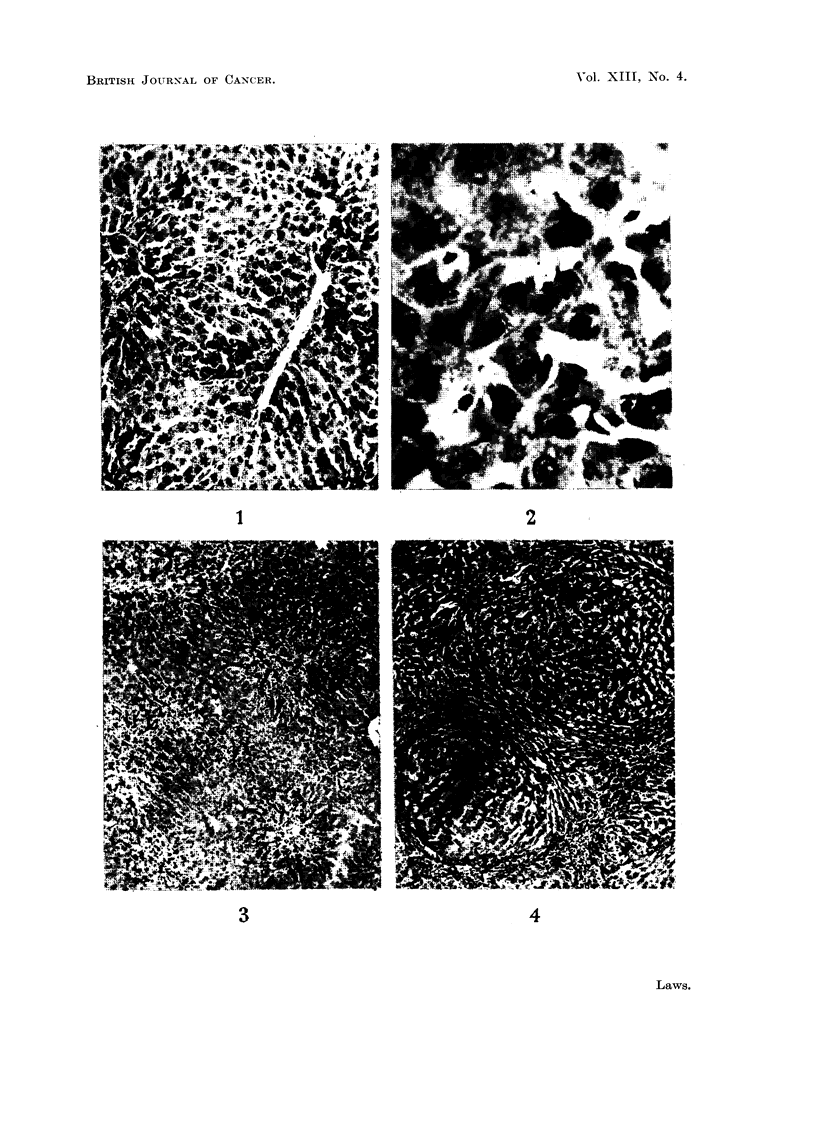

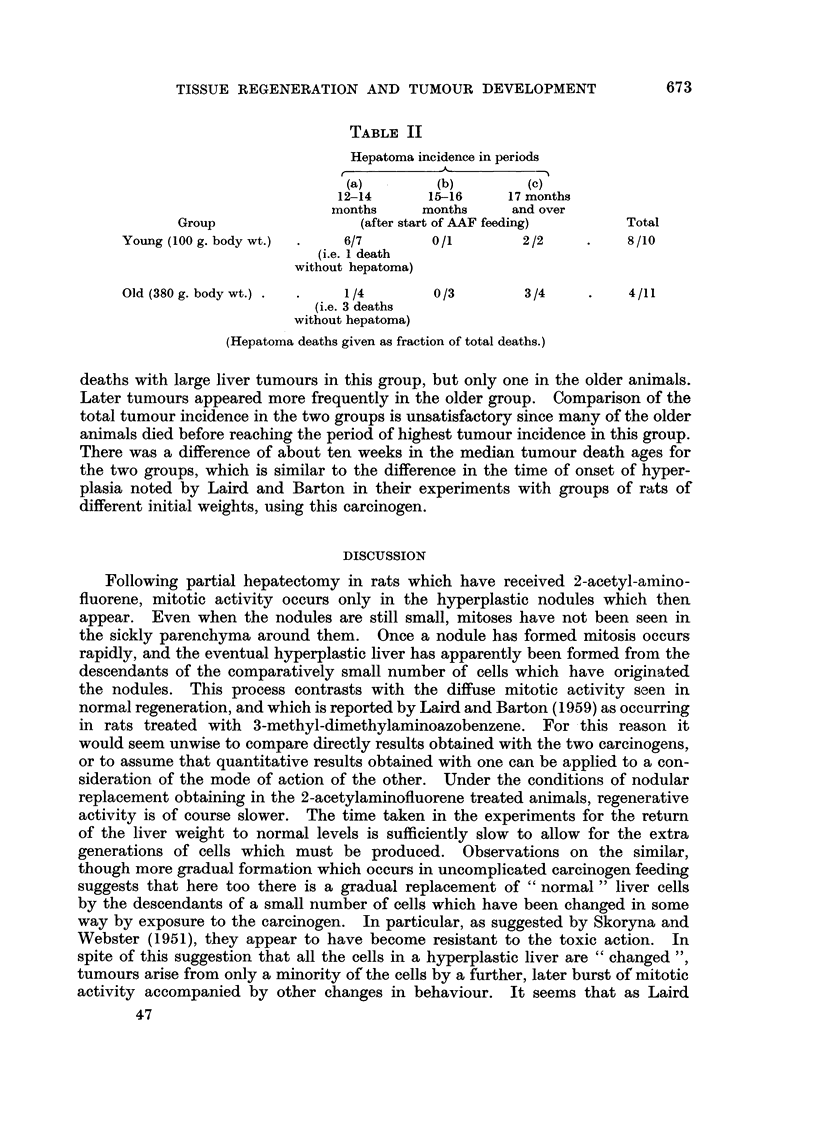

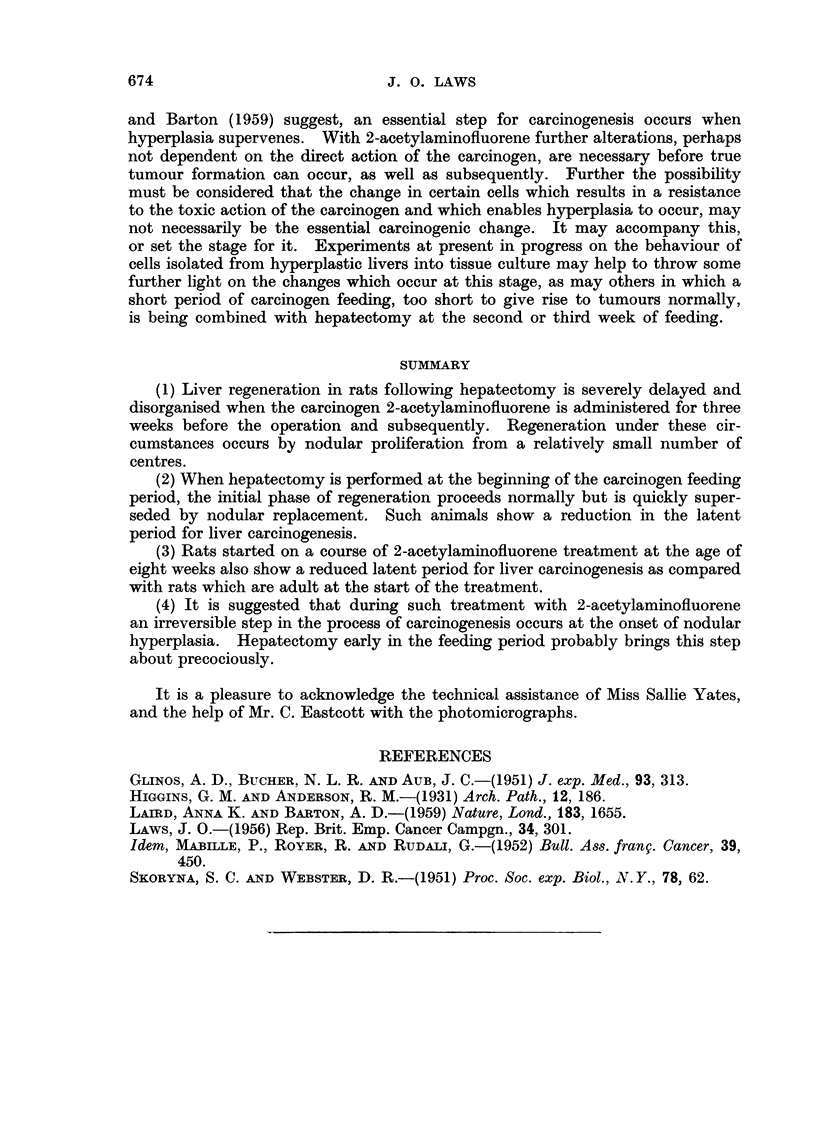


## References

[OCR_00362] GLINOS A. D., BUCHER N. L. R., AUB J. C. (1951). The effect of liver regeneration on tumor formation in rats fed 4-dimethylaminoazobenzene.. J Exp Med.

[OCR_00365] LAIRD A. K., BARTON A. D. (1959). Cell growth and the development of tumours.. Nature.

